# Factors Affecting the Variation in Sexual Activity and Response before and During Pregnancy among Pregnant Women in Rasht City, Northern Iran

**DOI:** 10.31661/gmj.v8i0.1531

**Published:** 2019-12-29

**Authors:** Shiva Alizadeh, Hedyeh Riazi, Hamid Alavi Majd, Giti Ozgoli

**Affiliations:** ^1^Student Research Committee, School of Nursing and Midwifery, Shahid Beheshti University of Medical Sciences, Tehran, Iran; ^2^Department of Midwifery and Reproductive Health, School of Nursing and Midwifery, Shahid Beheshti University of Medical Sciences, Tehran, Iran; ^3^Department of Biostatistics, School of Allied Medical Sciences, Shahid Beheshti University of Medical Sciences, Tehran, Iran

**Keywords:** Sexual Activity, Sexual Function, Pregnancy

## Abstract

**Background:**

Pregnancy is one of the most sensitive periods in a woman’s life, which sexual activity and intercourse are affected by the variations in physical, hormonal, and mental conditions. This study aimed to investigate the factors affecting the variations in sexual response before and during pregnancy.

**Materials and Methods:**

This cross-sectional study was conducted on pregnant women at Rasht city (northern Iran), 2018. The data were collected using the pregnancy sexual response inventory (PSRI). Statistical analysis was performed using descriptive and inferential tests by SPSS 25 at a significance level of P<0.05.

**Results:**

The mean total score of sexual activity and response of the subjects before and during pregnancy were 73.04 ± 14.81 and 46.88 ± 16.51, respectively. The variations in the total score of sexual activity and response during pregnancy decreased by 26.16 points during pregnancy compared to before pregnancy. There was a positive correlation between the number of children and the score of the variations in sexual activity and response before and during pregnancy (r=0.143).

**Conclusion:**

Couples with a higher level of education and a lower number of children had fewer variations in their sexual response. Therefore, it is possible to enhance the couples’ sexual health through encouraging them to appropriately plan for childbearing, to share the responsibilities of taking care of their children, and to continue their education at higher levels.

## Introduction

Sexual issues can be considered as important and rational aspects of an individual’s personal life and can be beyond mere sexual behavior. Sexual tendencies and behaviors are diverse and comprise of several factors. As pregnancy is one of the factors leading to physical and psychological changes in women, it can be regarded as an important element in the emergence or intensification of sexual disorders [1]. Pregnancy is one of the most sensitive periods in women’s lives, which sexual activity and intercourse are affected by changes in various physical, hormonal, social, cultural, and mental conditions [2-5]. Sexual activity and tendencies of pregnant women and their spouses are unpredictable during pregnancy and may increase, decrease, or remain unchanged [6]. Factors including physiological, anatomical, and hormonal changes; cultural, social, ethnic and religious issues; fears and concerns about sexual intercourse during pregnancy; changes in body image during pregnancy; and a sense of decrease in the physical attractiveness to spouse can all negatively affect the sexual response primarily in pregnant woman and then the sexual relationship between couples. These changes may result in anxiety and lack of confidence in couples; and eventually, disturb the mental health of the family [7]. Changes in the physical condition of pregnant women lead to changes in their sexual behaviors. During this period, women avoid some of their previous sexual behaviors [2, 4]. Besides, female sexual life during pregnancy can be affected by illnesses, gravidity, the number of children, gestational age, and beliefs, as well as pregnancy-related emotional and physical changes [8]. The normal sexual function of women including sexual arousal, lubrication, orgasm, and satisfaction leads to well-being and favorable quality of life [9]. Variations and decreased sexual function during pregnancy not only affect women but also have a negative effect on their sex partners/spouses, leading to low libido and thus affecting couples’ interactions [2, 10]. A healthy sexual condition during pregnancy is key to couples’ role as parents [7]. Therefore, an essential component in pregnant women’s healthcare examination is screening for sexual issues [6]. Regarding the importance of couples’ sexual health during pregnancy, some studies focusing on sexual function and response during pregnancy have only examined female sexual response, activity, and function during pregnancy [11-15].

While sexual intercourse involves both sexes, due to the female physical and mental state during pregnancy, spouses have a decisive role in improving sexual health during this particular period. The aforementioned aim can be achieved through examining the couples’ sexual function, activity, and satisfaction; along with their variations before and during pregnancy; as well as the factors that influence these measures. A study carried out in the city of Rasht, reported a high prevalence of sexual dysfunction during pregnancy [16]. Therefore, this study was conducted to investigate the factors affecting the variations in sexual response before and during pregnancy among women in Rasht city, Iran. The findings of this study help improve couples’ marital life during pregnancy by identifying and reinforcing the factors that positively affect sexual response.

## Materials and Methods

This cross-sectional study was performed on pregnant women. The total number of participants was 256, who were selected based on convenience sampling among pregnant women referred to the Prenatal Care Clinic, the State-run Hospital of Rasht city (northern Iran), from February to June 2018.

### Sample Size Calculation

The sample size was calculated using the following sample size formula with estimated standard error of 0.05 and based on previously reported prevalence of sexual dysfunction (60%) in pregnant women in Guilan province [16].

$$\begin{array}{l} \alpha =0.05\Rightarrow z_{1-\alpha /2}=1.96\\ p=0.60\\ n\geq \frac{z_{1-\alpha /2}^{2}(1-P)}{e^{2}P} \end{array}$$

### Inclusion and Exclusion Criteria

The inclusion criteria were women at 20 to 36 weeks gestation, being literate, absence of physiopathological illnesses, mental diseases, and marital problems and conflicts, based on self-report or medical records. Considering that the questionnaires were completed with the consent of the participants and by the individuals themselves, no questionnaire was excluded due to incomplete information, and no one withdrew from the study.

### Data Collection

Data were obtained via the Pregnancy Sexual Response Inventory (PSRI) tool, which also included questions regarding the demographic features of the subjects. The PSRI designed by Rudge *et al*. in Brazil, contains 26 questions about sexual activity and response in ten domains; the number of sexual intercourses, sexual desire, sexual arousal, orgasm, female sexual satisfaction, dyspareunia, sexual initiation, female sexual problems, male sexual satisfaction, and male sexual problems before and during pregnancy. The total score is calculated out of 100 points [17]. Scores below 25 indicate very bad sexual relations and activities, scores between 25 and 50 indicate bad sexual relations and activities, scores between 50 and 75 indicate good sexual relations and activities, and scores between 75 and 100 indicate excellent sexual relations and activities [18]. The English version of the PSRI questionnaire was translated into the Persian language after obtaining permission from the designer of the original questionnaire. The translation was performed by two of the researchers whose native language was Persian and had sufficient proficiency in English. Then, the Persian translate was reviewed and examined to ensure the accuracy of each item’s translation. The final Persian version of the instrument was developed considering the comments and the proposed equivalents by the experts. The Persian version was then back-translated into English by two translators (other than the initial ones) who were proficient in English and Persian and were not aware of the original English version of the questionnaire, the research, and its stages. After examining and modifying the two back-translated English versions, a single English version was developed. Finally, the English version was sent to the designer of the original questionnaire in order to make sure that the translation corresponded to the original questionnaire. The designer verified the questionnaire and sent the scoring system of the questionnaire to the researcher. The content, face, and construct validity of the questionnaire were also assessed. Briefly, 12 professors with adequate expertise and experience in the field as well as ten pregnant women who were eligible for the study were asked to examine the quality of the Persian version of the questionnaire in terms of grammatical correctness, appropriateness of the words, clarity, writing style, and spelling of the words. They were also asked to provide feedback to let the researchers correct formal and linguistic mistakes and resolve the ambiguities. Cronbach’s alpha coefficient and the test-retest methods were used to ensure the reliability and internal consistency of the whole questionnaire [19]. The reliability of the questionnaire was assessed based on the results of a pilot study on 30 pregnant women who were eligible for the study. The resultant Cronbach’s alpha coefficient was 0.891 for the questionnaires. An instrument can be considered reliable if the Cronbach’s alpha coefficient is equal to or greater than 0.7 [20-22]. Dilorio states that reliability is good if Cronbach’s alpha is higher than 0.8 [23]. The present questionnaire had excellent reliability due to the calculated Cronbach’s alpha. In order to examine the internal consistency and repeatability, the test-retest method was used. It is suggested that the interval between the two tests should be between two weeks to one month [20]. Therefore, the 30 pregnant women of the pilot study were asked to fill the questionnaire for the second time after two weeks, and the obtained scores in these two tests were compared using the intra-class correlation coefficient (ICC), which equaled 0.97. The ICC can be regarded as the most acceptable index for the consistency of tests [20].

### Ethical Considerations

All the steps taken in this study, including all the interactions with the human subjects, were in accordance with the ethical standards of Shahid Beheshti University of Medical Sciences; the 1964 Declaration of Helsinki and its later amendments; and general ethical standards. This study was approved by the Ethics Committee of the Shahid Beheshti University of Medical Sciences (code: IR.SBMU.PHNM.1395.495). The study was conducted after obtaining permission from the designer of the original questionnaire, providing the necessary explanations and information to the participants, and obtaining written consent from participants.

### Data Analysis

The data were analyzed via SPSS ver. 25 (SPSS Inc., Chicago IL, USA) statistical software. Descriptive statistics were used to describe the demographic data (the central indices, frequency, and percentage). Furthermore, student t-test and Pearson correlation coefficient were used at the significance level of P<0.05.

## Results

The mean age of the female participants was 28.46 ± 5.12 years; the minimum and maximum age of the participants were 13 and 42 years old, respectively. The mean gestational age of pregnant women was 30.83 weeks. Pregnancy in the majority of the subjects (84.8%) was intended. The level of education in most of the participants and their husbands was a high-school diploma or lower. The majority (73.4%) of the pregnant women were housewives. The demographic features of the participants are shown in Table-1. The mean the total sexual activity and response score of the participants before and during pregnancy were 73.04 ± 14.81 and 46.88 ± 16.51, respectively. The total score of the sexual activity and response during pregnancy decrease by 26.17 points compared to that of before pregnancy. Furthermore, the frequency of sexual activity was higher before pregnancy compared to the pregnancy period, and the sexual activity score showed a decrease of 39.77 points (Table-2). The Pearson correlation coefficient indicated no significant correlation between the score of the variations in sexual activity and response before and during pregnancy and the age of pregnant women (P=0.289), spousal age (P=0.248), couple age difference (P=0.828), length of marriage (P=0.225), gravidity (P=0.386), gestational age (P=0.092), and women’s occupation (P=0.295). However, there was a positive correlation between the score of the variations in sexual activity and response before and during pregnancy and the number of children (r=0.143, P=0.022). There was a negative correlation between the score of the variations in sexual activity and response before and during pregnancy and the pregnant women’s level of education (r=-0.174, P=0.005) and spouses’ level of education (r=-0.129, P=0.039, Table-3).

## Discussion

The results of this study indicated that the total score of sexual activity and response decreased during pregnancy compared to before pregnancy. Furthermore, the scores for the number of sexual intercourses, sexual desire, sexual arousal, orgasm, female sexual satisfaction, dyspareunia, sexual initiation, female sexual problems, male sexual satisfaction, and male sexual problems decreased during pregnancy compared to before pregnancy. In a study by Rudge *et al*. (2018) in Portugal, the PSRI was used. They found that the total score of sexual activity and response decreased during pregnancy compared to pre-pregnancy. They also found that the scores of all the domains, except for the dyspareunia, decreased during pregnancy compared to pre-pregnancy and that the mean score for dyspareunia during pregnancy was slightly lower than the mean score before pregnancy [18]. In a review study on the sexual behaviors of pregnant women, Jawed-Wessel *et al*. found a gradual decrease in the number of sexual intercourses from the first trimester of pregnancy to the third, and also a reduction of the number of sexual intercourses during pregnancy compared to pre-pregnancy [24]. The results of another study showed that most of the pregnant women had sexual intercourse once to thrice a month during pregnancy while they had sexual intercourse once or twice a week before pregnancy. Furthermore, with the increase in the gestational age, a decrease was observed in the number of sexual intercourses, mainly due to the reduction in sexual desire (35.5%), physicians’ recommendation (29%) and fear of injury to the fetus (29%). Fifty-four percent of the subjects in that study reported a decrease in sexual satisfaction during pregnancy compared to pre-pregnancy. One of the reasons for the reduction in sexual satisfaction was the decrease in the attractiveness of pregnant women [25]. Corbacioglu *et al*. [26] show that the number of women’s sexual intercourse decreased during pregnancy compared to pre-pregnancy. The decrease was more remarkable in the third trimester (76.5%). However, they found no significant relationship between the number of sexual intercourses before pregnancy and during pregnancy. There was also a decrease in the scores in all the domains of sexual response during pregnancy compared to pre-pregnancy [26]. In another study conducted by Yildiz *et al*. [27] in Turkey, the female sexual function index was used. They found that sexual function score decreased during pregnancy compared to pre-pregnancy and that the maximum decrease was observed in the third trimester [27]. In line with the present study, the results of some other studies indicated a reduction in sexual function during pregnancy compared to pre-pregnancy [12, 13, 15, 28-31]. Several pregnancy-related physiological and anatomical factors can reduce sexual activity in this period. Fatigue, backache, painful intercourse, infection (e.g., urinary tract infections, vaginitis, etc.), stress urinary incontinence, hemorrhoid, presence of the fetus in the pelvis, the instability of pubic symphysis and sacroiliac joints, nausea, and mastalgia are among the effective factors in reducing sexual activity and desire [2]. The relationship between socio-demographic variables and sexual response and function during pregnancy and their change compared to pre-pregnancy is a controversial issue. In the present study, there was no correlation between the score of the variations in sexual activity and response before and during pregnancy, and age of pregnant women, spousal age, age difference, duration of the marriage, gravidity, gestational age, and the occupation of women. However, there was a positive correlation between the score of the variations in sexual activity and response before and during pregnancy and number of children, and a negative correlation between the score of the variations in sexual activity and response before and during pregnancy and the level of education of pregnant women and their spouses. The results of the study conducted by Aydin *et al*. [28] indicated that the number of pregnancies and children had an adverse effect on sexual function in pregnancy. They found that sexual dysfunction during pregnancy increased with the increase in the number of pregnancies and children. But they failed to observe a significant relationship between sexual function and women’s age, number of abortions, and level of education in pregnant and non-pregnant women. Furthermore, they observed less sexual dysfunction among employed pregnant and non-pregnant women and women with a higher income [28]. Based on the results of the study conducted by Eryilmaz *et al*. [32], number of children negatively affects the number of sexual intercourses. Additionally, for women with higher levels of education, the number of sexual intercourse decreased during pregnancy compared with pre-pregnancy [32]. Contrary to the results of the studies discussed, the results of some studies showed that gravidity and number of children had no effect on the domains relating to pregnant women’s sexual response [15, 33]. Based on the results of present study and some of the mentioned studies on the positive correlation between the number of children and the variations in sexual response and dysfunction, it is possible to effectively increase the couples’ sexual function and satisfaction by encouraging them to appropriately plan for childbearing, and to share the responsibilities of taking care of their children. Anzaku *et al*. found that age affected orgasm in pregnant women, but had no effect on their sexual desire, arousal, and satisfaction [15]. The results of another study conducted in Turkey demonstrated that the age of pregnant women did not affect their sexual behavior and response [32]. However, the results of some studies indicate that age of pregnant women adversely affects sexual function during pregnancy [34, 35]. Pregnant women’s level of education affects their sexual function during pregnancy [34]. The results of the present study indicated that for men and women with a higher level of education, less variation could be detected in sexual response during pregnancy compared to pre-pregnancy. Similarly, the results of a study conducted in Egypt showed that women with lower levels of education were more likely to experience sexual dysfunction during pregnancy [33]. Nevertheless, the results of another study demonstrated that women’s higher level of education negatively affected their sexual satisfaction; however, it did not have an effect on other sexual domains [15]. It has been proven that people with a higher level of education have less sexual problems. It may be argued that the level of education, as one of the factors associated with personality, influences behavior [36]. Therefore, it seems that educated women are more interested in finding solutions to prevent sexual dysfunction during pregnancy. In the present study, no significant relationship was found between the women’s occupation and the variations in sexual response before and during pregnancy. Chang *et al*. [37] reported that full-time female employees had higher scores in sexual function and intercourse in the second trimester of pregnancy. However, the results of another study showed that the employed pregnant women had less sexual intercourses and that there was a significant negative relationship between employment and sexual intercourse, which may be due to work-related fatigue [38]. The results of another study conducted in Iran demonstrated that factors including older age, lower level of education, unintended pregnancy, and longer interval between marriage and pregnancy made women more prone to sexual dysfunction during pregnancy [16]. The discrepancies in the findings of the studies on the effects of demographic factors on pregnant women’s sexual response can be attributed to the diversity in research designs, the cultural diversity in female subjects geographic region, and the effects of social, cultural, and psychological factors as well as the couples’ relationship on the sexual relations of pregnant women in different communities. One of the strengths of this study is the use of a translated version of PSRI in a specific period in order to measure sexual response before and during pregnancy. Contrary to other questionnaires used in previous studies (e.g., FSFI), the PSRI questionnaire is a specialized instrument for examining the couples’ sexual activity and response during and before pregnancy. This questionnaire can be used by healthcare providers for assessing pregnant women’s sexual activity, response, and satisfaction and determining the necessity to refer them to a sexologist. Furthermore, healthcare providers can plan and implement strategic and health programs in order to improve pregnant women’s sexual health based on the scores in each domain. Some of the limitations of this study include the cross-sectional design and restriction to a specific geographical area. It is suggested that more studies be conducted in different geographic areas on participants with different cultures.

## Conclusion

The results of this study indicated that sexual activity and response decreased during pregnancy compared to pre-pregnancy. Besides, demographic factors, with the exception of the couples’ education and the number of children, did not affect the variations in sexual activity and response. The number of children had a positive correlation with the variations in sexual response during pregnancy compared to pre-pregnancy. The couples’ education had a negative correlation with the variations in sexual response before and during pregnancy. Thus, fewer children and a higher level of education can decrease the variations in the couples’ sexual response during pregnancy. Therefore, it is possible to enhance the couples’ sexual health and subsequently, to strengthen the family by encouraging them to plan appropriate childbearing, and to share the responsibilities of taking care of the children, and to continue their education at higher levels. Furthermore, sexual information should be provided to couples before and during pregnancy by healthcare providers in order to empower women to maintain and improve their sexual health.

## Acknowledgment

The present study was part of a doctoral dissertation in the field of Reproductive Health, approved by the Shahid Beheshti University of Medical Sciences and was approved by the university Ethics Committee (code IR.SBMU.PHNM.1395.495). The authors hereby thank the university officials and the midwives at the Prenatal Care Clinic, and all the participants in this study.

## Conflict of Interest

The authors declare no conflict of interest.

**Table 1:** Demographic Features of the Subjects

**Characteristics**	**Mean±SD or n(%)**	**Min-Max**
**Age, y**		
Pregnant woman	28.46 ± 5.12	13 – 42
Spouse	32.36 ± 4.73	21 - 45
**Gravidity**	1.71 ± 0.74	1 – 5
**Number of children**	0.54 ± 0.58	0 – 2
**Gestational age**	30.83 ± 3.63	20 – 35
**Duration of marriage, y**	6.44 ± 4.24	1 – 20
**Age difference, y**	4.02 ± 2.87	0 – 16
**Pregnancy**		
Intended	217 (84.8)	-
Unintended	39 (15.2)	-
**Abortion history**		
Yes	48 (18.8)	-
No	208 (81.3)	-
**Education level**		
Diploma or lower	163 (63.7)^a^, 165 (64.5)^b^	-
Post-diploma or Bachelor	84 (32.8)^a^, 81 (31.6)^b^	-
Master’s degree or Ph.D	9 (3.5)^a^, 10 (3.9)^b^	-
**Occupation status**		
Housewife^a^	188 (73.4)	-
Employed^a^	68 (26.6)	-
Office worker^b^	50 (19.5)	-
Self-employed^b^	190 (74.2)	-
Other^b^	16 (6.3)	-

**a** Pregnant woman **b** Spouse

**Table 2:** The Scores of the Subjects’ Sexual Activity and Response

**Questions**	**Mean ± SD**	**95% Confidence interval**
**The total score of sexual activity and response before pregnancy**	73.04 ± 14.81	(71.22, 74.86)
**The total score of sexual activity and response during pregnancy**	46.88±16.51	(44.84, 48.90)
**Variations in the total score of sexual activity and response before and during pregnancy**	- 26.17 ± 19.45	(-28.56, -23.77)
**Variations in the score of the number of sexual intercourses before and during pregnancy**	- 39.77 ± 30.54	(-43.53, -36.02)
**Variations in the score of sexual desire before and during pregnancy**	- 9.17 ± 48.29	(-15.12, -3.24)
**Variations in the score of sexual arousal before and during pregnancy**	- 20.11 ± 35.36	(-24.47, -15.76)
**Variations in the score of orgasm before and during pregnancy**	- 25.78 ± 35.41	(-30.14, -21.42)
**Variations in the score of female sexual satisfaction before and during pregnancy**	- 34.17 ± 34.42	(-38.41, -29.94)
**Variations in the score of dyspareunia before and during pregnancy**	- 10.15 ± 45.74	(-15.79, -4.53)
**Variations in the score of sexual initiation before and during pregnancy**	- 6.44 ± 27.43	(-9.82, -3.07)
**Variations in the score of female sexual problems before and during pregnancy**	- 15.62 ± 43.27	(-20.96, -10.30)
**Variations in the score of male sexual satisfaction before and during pregnancy**	- 27.93 ± 42.14	(-33.12, -22.74)
**Variations in the score of male sexual problems before and during pregnancy**	- 8.59 ± 33.20	(-12.68, -4.51)

**Table 3:** The Correlation Coefficient Between Demographic Characteristics and the Scores of Activity Change and Sexual Response.

**Characteristics**	**P-value**	**Correlation (r)**
**Age at pregnancy**	0.289	-0.066
**Spouse’s age**	0.248	-0.073
**Age difference**	0.828	0.014
**Marriage duration**	0.225	-0.076
**Gravidity**	0.386	0.054
**Gestational age**	0.092	-0.106
**Woman’s occupation**	0.295	-0.038
**Number of children**	0.022	0.143*
**Woman’s level of education**	0.005	-0.174**
**Spouse’s level of education**	0.039	-0.129*

* Correlation is significant at the 0.05 ** Correlation is significant at the 0.01
